# A Genetic Resource for Rice Improvement: Introgression Library of Agronomic Traits for All AA Genome *Oryza* Species

**DOI:** 10.3389/fpls.2022.856514

**Published:** 2022-03-24

**Authors:** Yu Zhang, Jiawu Zhou, Peng Xu, Jing Li, Xianneng Deng, Wei Deng, Ying Yang, Yanqiong Yu, Qiuhong Pu, Dayun Tao

**Affiliations:** Yunnan Key Laboratory for Rice Genetic Improvement, Food Crops Research Institute, Yunnan Academy of Agricultural Sciences, Kunming, China

**Keywords:** rice, AA genome, introgression line, grain size, allelic variation

## Abstract

Rice improvement depends on the availability of genetic variation, and AA genome *Oryza* species are the natural reservoir of favorable alleles that are useful for rice breeding. To systematically evaluate and utilize potentially valuable traits of new QTLs or genes for the Asian cultivated rice improvement from all AA genome *Oryza* species, 6,372 agronomic trait introgression lines (ILs) from BC_2_ to BC_6_ were screened and raised based on the variations in agronomic traits by crossing 170 accessions of 7 AA genome species and 160 upland rice accessions of *O. sativa* as the donor parents, with three elite cultivars of *O. sativa*, Dianjingyou 1 (a *japonica* variety), Yundao 1 (a *japonica* variety), and RD23 (an *indica* variety) as the recurrent parents, respectively. The agronomic traits, such as spreading panicle, erect panicle, dense panicle, lax panicle, awn, prostrate growth, plant height, pericarp color, kernel color, glabrous hull, grain size, 1,000-grain weight, drought resistance and aerobic adaption, and blast resistance, were derived from more than one species. Further, 1,401 agronomic trait ILs in the Dianjingyou 1 background were genotyped using 168 SSR markers distributed on the whole genome. A total of twenty-two novel allelic variations were identified to be highly related to the traits of grain length (GL) and grain width (GW), respectively. In addition, allelic variations for the same locus were detected from the different donor species, which suggest that these QTLs or genes were conserved and the different haplotypes of a QTL (gene) were valuable resources for broadening the genetic basis in Asian cultivated rice. Thus, this agronomic trait introgression library from multiple species and accessions provided a powerful resource for future rice improvement and genetic dissection of agronomic traits.

## Introduction

Rice is one of the most important staple crops for almost half of the world’s population. The Food and Agriculture Organization of the United Nations predicts that rice yield will have to be increased 50 to 70% by 2050 to meet human’s demands, which increases that rice yield is still central for maintaining global food security (Ray et al., 2013). Whereas rice yield potential has been stagnant since the introduction of semidwarf gene into cultivated rice and the utilization of heterosis (Virmani et al., 1982; Monna et al., 2002; Sasaki et al., 2002), the narrow genetic basis that results from the overuse of few parental materials and the lack of favorable variations led to yield bottleneck in rice breeding (Tanksley and Mccouch, 1997).

Genus *Oryza* contains twenty-two wild species and two cultivated rice species that represent 11 genomes: AA, BB, CC, BBCC, CCDD, EE, FF, GG, HHJJ, HHKK, and KKLL (Khush, 1997). Among these, six wild rice species (*O. nivara*, *O. rufipogon*, *O. barthii*, *O. glumaepatula*, *O. longistaminata*, and *O. meridionalis*) and two cultivated species (*O. sativa* and *O. glaberrima*) were classified into the AA genome. Asian cultivated rice (*O. sativa* L.) was domesticated from wild species *O. rufipogon* thousands of years ago (Khush, 1997; Huang et al., 2012). Previous reports indicated that 40% of the alleles of *O. rufipogon* was lost during the domestication from common wild rice to the cultivated rice (Sun et al., 2002), and only 10–20% of the genetic diversity in *O. rufipogon* and *O. nivara* was retained in two subspecies of the cultivated rice (Zhu et al., 2007). Since sharing the same AA genome, *O. glaberrima* and the six wild rice species are the most accessible gene pool for rice improvement (Ren et al., 2003). Thus, the exploitation and utilization of the useful alleles of AA genome species may overcome yield plateaus of *O. sativa* (Xiao et al., 1998). However, it is difficult to utilize the natural genetic diversity because of reproductive isolation, linkage drag, and background noise. Moreover, many important agronomic traits that include yield are controlled by quantitative trait loci (QTL) with smaller effect, which can be influenced by the environment. It is difficult to understand the QTL-controlled agronomic traits because of their complex inheritance and the genetic background noise.

Introgression lines are genetic resource in which the whole genome of a donor genotype (DG) is represented by the different segments in the genetic background of elite varieties. Genetic background noise of ILs can be eliminated significantly, which can be evaluated for any traits’ improvement over the recurrent parents for rice breeding, also for QTL mapping and gene discovering as a single Mendelian factor; in addition, potential favorable genes hidden in the background of related species could be expressed in the genetic background of cultivated rice (Ballini et al., 2007; Eizenga et al., 2009; Bian et al., 2010; Rama et al., 2015; Jin et al., 2016; Yang et al., 2016; Bhatia et al., 2017; Bhatia et al., 2018; Yamagata et al., 2019). Thus, ILs that eliminate hybrid sterility, linkage drags, and background noise are one of the most important genetic resources for QTL mapping, gene identification, and discovery and rapid utilization for commercial breeding. Though a series of introgression lines developed by the genome-wide marker selections were obtained from the intersubspecific crosses between *indica* and *japonica* varieties and from the interspecific crosses between Asian cultivated rice and wild relatives of *Oryza sativa* (Bhatia et al., 2017; Divya et al., 2019), some lines showed the remarkable phenotype, whereas others did not exhibit obvious agronomic traits, which were difficult to be used for QTL identification, gene cloning, and breeding improvement. Establishing the introgression lines based on agronomic trait selection might be time-consuming and less laborious strategy.

AA genome species distributed in the natural and wild environment, which contains amount of useful allelic genes for improving rice yield and resistance to biotic and abiotic stresses (Khush, 1997). Different AA genome wild species and native varieties with unique characteristics and ecological adaptability represented the independent center of genetic diversity in rice. Comprehensively and systemically developing the ILs from all the AA genome species in different elite cultivar varieties background will help us to achieve sustainable yield improvement, diverse requirements for quality, and broad-spectrum resistance so as to meet the demand of the modern breeding program.

In this study, to explore and utilize wild relatives in rice improvement, we systematically introduced foreign segments from eight different AA genome species (*O. longistaminata*, *O. barthii*, *O. glumaepatula*, *O. meridinalis*, *O. nivara*, *O. rufipogon*, *O. glaberrima*, and upland rice of *O. sativa*) into three elite, highly productive *O. sativa* varieties (Dianjingyou 1, Yundao 1 and RD23). A total of six thousand three hundred and seventy-two agronomic ILs in three different backgrounds were screened and developed based on the repeated evaluation and selection of agronomic traits. One thousand four hundred and one of 6,372 agronomic ILs in the Dianjingyou 1 background were used to analyze genotype and discover novel alleles for grain size. Thus, this agronomic introgression library provided a powerful resource for future rice improvement and genetic dissection of agronomic traits.

## Materials and Methods

### Plant Materials

The plant materials included 1 accession of *O. longistaminata*, 13 accessions of *O. barthii*, 6 accessions of *O. glumaepatula*, 8 accessions of *O. meridionalis*, 19 accessions of *O. rufipogon*, 20 accessions of *O. nivara*, 103 accessions of *O. glaberrima*, and 160 upland rice varieties of *O. sativa* (Supplementary Table 1). Three elite varieties, Dianjingyou 1 (a *japonica* variety), Yundao 1 (a *japonica* variety), and RD23 (an *indica* variety), were used as the recurrent parents.

A total of three hundred and twenty-nine accessions of AA genome species as the donor parents except for *O. longistaminata* were crossed with Dianjingyou 1 as the recurrent parent. A total of two hundred and twenty-six accessions as the donor parents, except for *O. longistaminata* and *O. glaberrima*, were used to cross with the recurrent parent Yundao 1. All the F_1_ plants were used as female parents to backcross to their respective recurrent parents to produce BC_1_F_1_ generation. More than 200 BC_1_F_1_ seeds were generated for each of the combinations. The moderate heading date of individuals was selected to backcross with the recurrent parents, and about 200 BC_2_F_1_ seeds were obtained. From each of the BC_2_F_1_ progeny, individuals that showed a significant agronomic difference from the recurrent parents were selected for further backcrossing or selfing. After 2–6 times backcrossing and 2–7 times selfing, the progeny with stable and different target traits from their recurrent parents was developed as agronomic ILs.

The F_1_ plants were obtained by embryo rescue technique from the cross between 1 accession of *O. longistaminata* as the donor parent and an *indica* variety RD23 as the recurrent parent, and crossing and selfing from BC_1_F_1_ generation were performed as above mentioned procedure.

All materials were grown at the Sanya Breeding Station, Sanya (18.24° N, 109.50° E), Hainan province, China. Ten individuals per row were planted at a spacing of 20 cm × 25 cm. All materials were grown and managed according to the local protocol.

### Agronomic Trait Evaluation

A randomized complete block design was carried out with three replications for agronomic trait evaluation under two different environments (E1: winter and dry season, December to April 2008–2009; E2: summer and rainy season, July to November 2009), respectively. Each line was planted in three rows with 10 individuals per row. The five plants in the middle of each row were used for scoring traits. The recurrent parents, Dianjingyou 1, Yundao 1, and RD23, were used as controls in the experiment, respectively.

Prostrate growth habit was observed for the tiller angle in three main stages, which includes booting stage, heading stage, and grain filling stage. That tiller angle in ILs was larger than that in recurrent parent, which was regarded as the prostrate growth.

Primary branches at the base of panicle of the lines extend outward were regarded as the spreading panicle. Erect panicle or drooping panicle was evaluated according to the angle between the lines that connecting panicle pedestal with panicle tip and the elongation line of stem; spikelet numbers were measured as the total number of spikelets of the whole plant divided by its total number of panicles. Dense panicle was scored by the ratio of spikelet numbers to panicle length.

Tiller number was recorded from five random plants; plant height was measured from the ground level to the tip of the tallest panicle.

To measure the grain size, grains were selected from primary panicle and stored at room temperature for at least 3 months before testing. Twenty grains were used to measure grain length (GL), grain width (GW), and the ratio of grain length to grain width (RLW) from each plant. Photographs of grains per individual were taken using stereomicroscope, and then, grain size was measured by software Image J. The average value of 20 grains was used as phenotypic data. The weight of one thousand grains was measured by weighting fertile, fully mature grains from five panicles.

Aerobic adaptation was evaluated by biomass, yield, harvest index, heading date, and plant height in both aerobic and irrigated environments. Drought tolerance was assessed by the same traits as the aerobic adaptation in both upland and irrigated environments. For the aerobic and upland treatments, we used direct sowing with 4 seeds per hole and retained one seedling at the three-leaf stage. There is some difference in water management, and the rainfall provided the essential water for plant growth without the extra irrigation under aerobic treatment, whereas mobile sprinkler irrigation facilities were used to maintain a humid soil environment at the sow, tiller, and heading stage under upland treatment. For the irrigating treatment, sowing and transplanting single seedlings were done, and the field was managed according to the local standard practices.

To evaluate blast resistance, introgression lines were inoculated with *Magnaporthe oryzae* for 3 weeks after sowing by spraying with conidial suspension. After 7 days, lesion types on rice leaves were observed and scored according to a standard reference scale based on a dominant lesion type (Xu P. et al., 2015).

For a simply inherited trait, awn, pericarp color, and kernel color were observed directly in the field.

### DNA Extraction and PCR Protocol

The experimental procedure for DNA extraction was performed as previously described (Edwards et al., 1991); A total of 168 SSR markers were selected from the Gramene database^1^ or previously published polymorphic SSR markers within the *Oryza* AA genome species (McCouch et al., 2002; Orjuela et al., 2010). PCR was performed as follows: a total volume of 10 μl containing 10 ng template DNA, 1 × buffer, 0.2 μM of each primer, 50 μM of dNTPs, and 0.5 unit of Taq polymerase (Tiangen Company, Beijing, China). The reaction mixture was incubated at 94°C for an initial 4 min, followed by 30 cycles of 94°C 30 s, 55°C 30 s, and 72°C 30 s, and a final extension step of 5 min at 72°C. PCR products were separated on 8% non-denaturing polyacrylamide gel and detected using the silver staining method.

### Determination of the Length of a Substituted Segment in Introgression Lines

The substituted segment was counted based on the SSR markers distributed on twelve chromosomes (Paterson et al., 1988; Young and Tanksley, 1989; McCouch et al., 2002). Intervals between two markers homozygous for the DG were regarded as 100% introgression segment, and a chromosome segment flanked by one marker of the DG and one marker of the recurrent type (DR) was considered as 50% introgression segment, whereas intervals between two markers homozygous for the recurrent genotype (RG) represented the background genotype. Thus, the physical distance of both DD and half of DR was used to estimate the length of introgression segments. The expected introgression length of the genome is divided by the total genome size to yield the expected proportion of introgression.

### Exploring Loci for Grain Size Based on the Introgression Lines

Statistical analyses were performed on the SAS software package. The linkage between loci and grain size was scored by binomial distribution based on the genotype and phenotype between ILs and the recurrent parent. The genotypes of ILs that showed a significant difference from the recurrent parent in grain size were used to perform QTL analysis, if the rate of DG at some loci was significantly higher than that of the theoretical prediction, this locus could be linked with the grain size. Significant level was determined by the comparison between the ILs and the recurrent parents. Dunnett’s *t*-test at *p* < 0.0001 was set to decrease the false probability (Eshed and Zamir, 1995; Xu et al., 2014).

## Results

### Agronomic Trait Diversity in Introgression Library From AA Genome Donors

**FIGURE 1:**
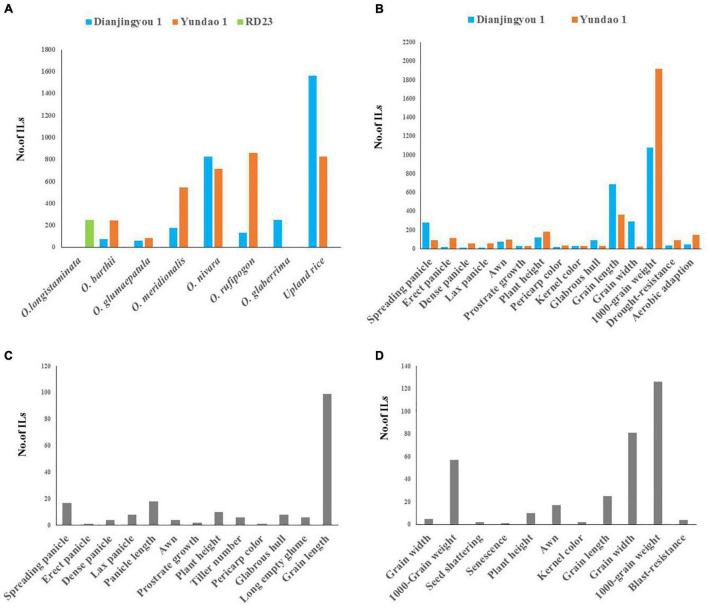
The summary of introgression libraries with the donor of 8 AA genome species in three different genetic backgrounds. **(A)** A number of ILs from different donors in the Dianjingyou 1, Yundao 1, and RD23 background. **(B)** A number of ILs derived from *O. barthii*, *O. glumaepatula*, *O. meridionalis*, *O. nivara*, *O. rufipogon*, and upland rice, respectively, showed agronomic traits distinguishing from their recurrent parents Dianjingyou 1 and Yundao 1. **(C)** Agronomic traits of ILs derived from crosses between *O. glaberrima* and Dianjingyou 1. **(D)** Agronomic traits of ILs derived from cross between *O. longistaminata* and RD23.

To systematically explore potentially valuable genes hidden in the AA genome wild relatives and two cultivated species, 67 accessions of AA genome wild rice, 103 accessions of *O. glaberrima*, and 160 upland rice varieties of *O. sativa* as the donors were used to raise the agronomic introgression line library. Of these accessions, all accessions except for *O. longistaminata* were used for generating ILs in the Dianjingyou 1 background, 233 donors except for the accessions of *O. glaberrima* and *O. longistaminata* were used for developing ILs in the background of Yundao 1, and 1 accession of *O. longistaminata* was used for raising ILs in the RD23 background. A total of 6,372 introgression lines with multiple donors showed a remarkable difference in the agronomic traits, which includes spreading panicle, erect panicle, dense panicle, lax panicle, awn, prostrate growth, plant height, pericarp color, kernel color, glabrous hull, grain size, 1,000-grain weight, drought resistance, and aerobic adaption, compared with their recurrent parents (Figures 1A–D). Among these, 74, 61, 179, 824, 135, 251, and 1561 ILs that show distinguished traits in the Dianjingyou 1 background were selected from the donors of *O. barthii*, *O. glumaepatula*, *O. meridionalis*, *O. rufipogon*, *O. nivara*, *O. glaberrima*, and upland rice of *O. sativa*, respectively (Figures 1A,C and Supplementary Tables 2, 3). Additionally, 244, 85, 547, 714, 858, and 825 ILs that exhibit different agronomic traits in the Yundao 1 background were developed from the donors of *O. barthii*, *O. glumaepatula*, *O. meridionalis*, *O. rufipogon*, *O. nivara*, and upland rice of *O. sativa*, respectively (Figure 1A and Supplementary Table 4). A total of two hundred and sixty-five ILs were derived from the cross between 1 accession of *O. longistaminata* as the donor and an *indica* variety RD23 as the recurrent parent (Figure 1D and Supplementary Table 5). Thus, the agronomic introgression library derived from the multiple donors of AA genome species in the different backgrounds showed the abundant genetic variations in the agronomic traits.

For the same donor parent, phenotype variations for the agronomic traits varied with the genetic background. The numbers of ILs that show erect panicle, dense panicle, lax panicle, awn, plant height, pericarp color, 1,000-grain weight, drought-resistance, and aerobic adaption in Yundao 1 background was more than those of Dianjingyou 1 background, whereas the number of ILs that exhibit spreading panicle, prostrate growth, kernel color, glabrous hull, GL, and GW in Yundao 1 background was less than those of Dianjingyou 1 background (Figure 1B). It suggested that target trait expression was depended on the background of recurrent parent difference to a certain degree. Developing an introgression library in the different backgrounds will be beneficial to express hidden genes in the donor and discover more genetic variations for further study.

### Characteristics of Chromosome Substituted Segments in the Introgression Library

A total of 168 SSR markers distributed on 12 chromosomes were selected to genotype agronomic introgression library in Dianjingyou 1 background (Supplementary Figure 1). The length of the interval between two markers ranged from 0.2 to 5.5 Mb, with an average of 2.22 Mb on the rice physical map (Table 1 and Supplementary Figure 1). The polymorphism rate displayed from 82.74 to 98.43% between seven AA genome species and Dianjingyou 1 (Table 1).

**TABLE 1:** The description of markers used for genotyping introgression library.

Chr	Number of Markers	Density (Mb)	The marker polymorphism rate (%)						
			*O. barthii*	*O. glumaepatula*	*O. meridionalis*	*O. nivara*	*O. rufipogon*	*O. glaberrima*	Upland rice
1	17	2.55	94.12	94.12	100.00	100.00	100.00	100.00	100
2	18	2.00	77.78	77.78	88.89	83.33	88.89	94.44	77.78
3	16	2.28	100.00	100.00	100.00	100.00	100.00	100.00	100.00
4	15	2.37	80.00	100.00	93.33	100.00	100.00	100.00	93.33
5	15	2.00	93.33	60.00	86.67	93.33	93.33	86.67	80.00
6	13	2.40	92.31	84.62	100.00	100.00	100.00	100.00	100.00
7	14	2.12	92.86	78.57	92.86	92.86	92.86	100.00	85.72
8	14	2.03	100.00	57.14	100.00	100.00	100.00	100.00	78.58
9	10	2.30	100.00	90.00	100.00	100.00	100.00	100.00	83.34
10	12	1.93	91.67	100.00	100.00	100.00	91.67	100.00	100.00
11	12	2.42	66.67	66.67	83.33	100.00	83.33	100.00	100.00
12	12	2.29	83.33	83.33	100.00	100.00	100.00	100.00	100.00
Mean (%)		2.22	89.29	82.74	95.24	97.02	95.83	98.43	91.56

A total of one thousand four hundred and one IL in the Dianjingyou 1 background were used to detect the characteristics of chromosome segments from seven AA genome species, which include 29 ILs from *O. barthii*, 30 ILs from *O. glumaepatula*, 76 ILs from *O. meridionalis*, 380 ILs from *O. nivara*, 74 ILs from *O. rufipogon*, 81 ILs from *O. glaberrima*, and 731 ILs from upland rice of *O. sativa* (Supplementary Table 6). In the 29 ILs from the donor of *O. barthii*, the length of introgression segments ranged from 2.66 to 28.98 Mb, averaging 6.99 Mb (Table 2). Different coverage rate was observed in a different chromosome. Chromosomes 3, 8, and 9 had 100% coverage rate, whereas chromosome 11 only had 39.14% coverage rate (Supplementary Figure 2 and Supplementary Table 7).

**TABLE 2:** The characterization of introgression lines.

Donors	Number of introgression lines	Number of introgression Segments	Average number of segments per chromosome	Range of Segment length (Mb)	Average length of segments (Mb)	Average background recovery rate(%)
*O. barthii*	29	349	29.08	2.66–28.98	6.99	88.92
*O. glumaepatula*	30	210	17.50	0.66–25.60	5.35	91.94
*O. meridionalis*	76	434	36.17	0.27–23.77	5.83	93.21
*O. nivara*	380	3332	277.67	0.30–27.43	5.84	92.74
*O. rufipogon*	74	625	52.08	0.28–23.46	6.37	89.92
*O. glaberrima*	81	1138	94.83	0.27–24.17	5.80	85.14
Upland rice	731	9198	766.50	0.19–43.27	6.67	84.03

A total of thirty ILs were obtained from an interspecific backcross between the cultivated rice *O. sativa* Dianjingyou 1 and the wild relative *O. glumaepatula*, and the length of introgression segments ranged from 660 kb to 25.6 Mb, averaging 5.35 Mb (Table 2). The ILs covered 73.11% of *O. glumaepatula* genome in the Dianjingyou 1 background (Supplementary Figure 3 and Supplementary Table 8).

In the Dianjingyou 1/*O. meridionalis* introgression library, the length of introgression segments was detected from 0.27 to 23.77 Mb, averaging 5.83 Mb (Table 2). The donor introgressions covered 89.17% *O. meridionalis* genome. Chromosomes 3, 6, and 8 exhibited complete coverage, whereas chromosome 11 showed the least coverage rate of 60.71% (Supplementary Figure 4 and Supplementary Table 9).

A total of three hundred and eight ILs were developed with the donor of *O. nivara*, and the average length of introgression segments was 5.84 Mb (Table 2). Average coverage rate per chromosome was about 97.17% (Supplementary Figure 5 and Supplementary Table 10).

In the introgression library with the donor of *O. rufipogon*, the length of introgression segments varied from 280 kb to 23.46 Mb, averaging 6.37 Mb, and the average number of segments per chromosome was 52.08 (Table 2). Chromosomes 3, 8, and 9 exhibited 100% coverage rate, whereas chromosome 2 showed the least coverage rate of 74.43% (Supplementary Figure 6 and Supplementary Table 11).

We developed 81 ILs with the *O. glaberrima* species as the donor, and the length of introgression segments varied from 270 kb to 24.17 Mb, averaging 5.80 Mb (Table 2). Chromosomes 3, 6, 9, and 11 exhibited complete coverage, whereas chromosome 10 showed the least coverage rate of 78.65% (Supplementary Figure 7 and Supplementary Table 12).

In the introgression library derived from the donor of upland rice in *O. sativa*, the average length of introgression segments was 6.67 Mb (Table 2). All the chromosomes except for chromosome 10 showed 100% upland rice genome coverage (Supplementary Figure 8 and Supplementary Table 13).

Taken together, ILs covered 81.19%, 73.11%, 89.17%, 97.17%, 89.19%, 90.70%, and 99.10% of *O. barthii*, *O. glumaepatula*, *O. meridionalis*, *O. nivara*, *O. rufipogon*, *O. glaberrima*, and upland rice genome information, respectively, which suggests that systematic and comprehensive agronomic IL library with the donor of AA genome species was developed by agronomic trait selection.

### Detection of Potential Allelic Variations for Grain Size in the Introgression Library

**FIGURE 2:**
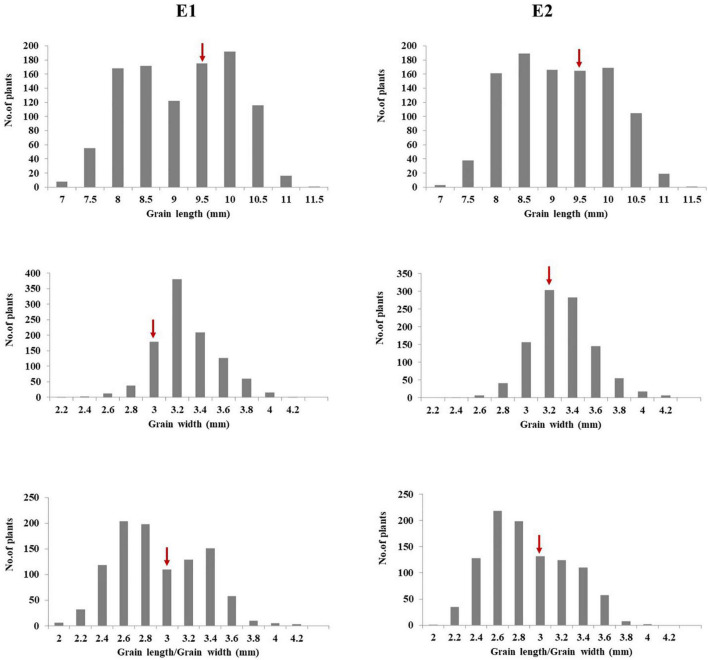
Frequency distribution of grain size traits in introgression lines in the Dianjingyou 1 background. Arrows indicated the mean values for recurrent parent Dianjingyou 1.

Seed size plays an important role in rice yield (Xing and Zhang, 2010). Grain size not only determines rice appearance, but also affects milling, cooking, and eating quality of rice (Fan et al., 2006). Significant variations were observed for GL, GW, and the RLW in the introgression library with multiple donors in the background of Dianjingyou 1. Some ILs for GL, GW, and RLW were found to be significantly superior to the recurrent parent Dianjingyou 1. For GL, 133 and 125 ILs were found to be significantly longer than the Dianjingyou 1 in two seasons, respectively. For GW, 412 and 508 ILs were observed to be significantly wider than the recurrent parent in two environments, respectively. For the RLW, 277 and 178 ILs were found to be higher than the Dianjingyou 1 in different seasons, respectively (Figure 2). In addition, the same traits in the different environments showed a highly significant correlation (Supplementary Table 14). These results suggested that abundant genetic variations for grain size existed in the wild and cultivated accessions of rice.

**FIGURE 3:**
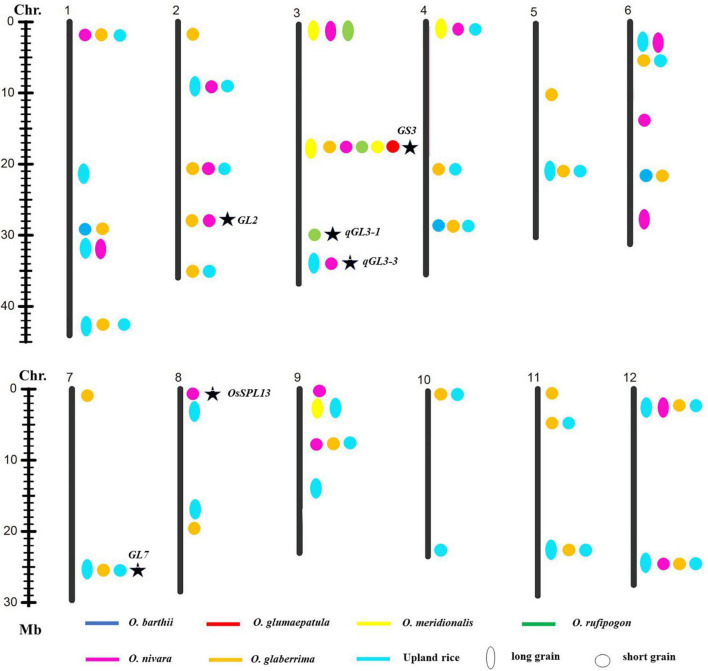
Allelic variation linking with GL was detected from the different donors. Asterisk indicated published genes.

**FIGURE 4:**
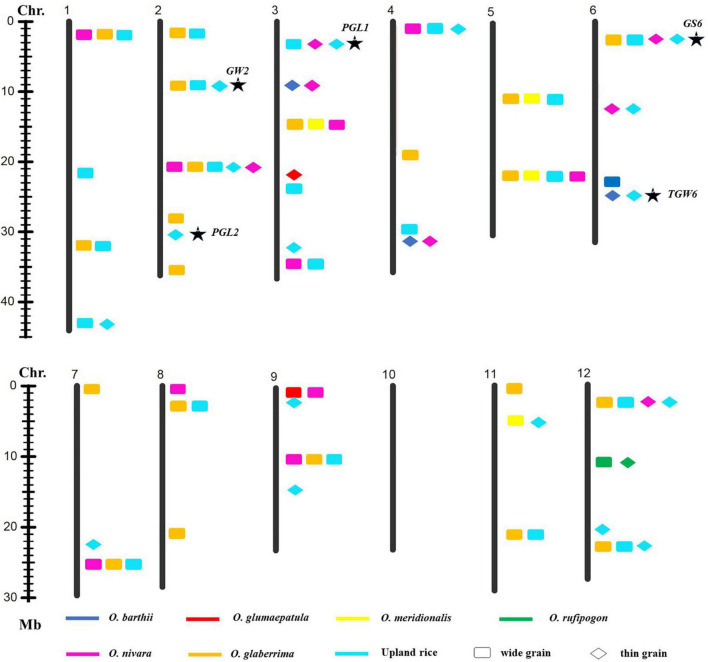
Allelic variation linking with GW was detected from the different donors. Asterisk indicated published genes.

**FIGURE 5:**
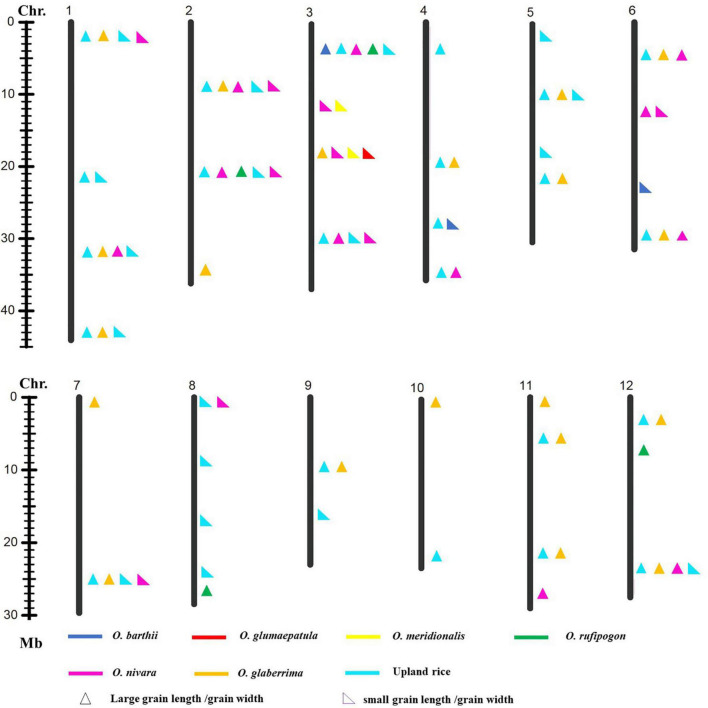
Allelic variation linking with the RLW was detected from the different donors.

To explore favorable allelic variation for grain size, QTLs were detected based on the genotype and phenotype data. A total of forty-one loci linking with GL, forty-four loci linking with GW, and thirty-two loci linking with RLW were identified. It indicated that abundant gene pool for grain size existed in the AA genome species (Figures 3–5). Among these, 26 loci for GL were detected from multiple donors, and 12, 11, 2, and 1 loci were found from the donors of two species, three species, four species, and six species, respectively. It suggested that the same locus that contributes to GL is a potential allelic variation from different donors. Moreover, 4 loci from the different donors were only responsible for long grain, 12 loci derived from the multiple donors only contributed to short grain, and 10 loci from the different species controlled both long grain and short grain. Moreover, 22 novel allelic variations from multiple donors contributed to GL (Figure 3).

A total of 27 loci for GW were examined from the multiple donors, which include 13 loci from the only two species, 9 loci from three species, 3 loci from four species, and 1 locus from five species (Figure 4). Moreover, 12 loci from the different donors were only responsible for wide grain, 4 loci from multiple species only led to thin grain, and 10 loci from the different species controlled both wide and thin grains. In addition, 22 novel allelic variations for GW were found in agronomic IL library (Figure 4).

Nineteen loci for RLW from multiple donors were explored on 12 chromosomes, which include 12, 6, and 1 locus detected simultaneously in two, three, and four donor species, respectively (Figure 5).

These results indicated that detection of favorable genes using multiple donors could help us to find the novel allelic variations. The allelic genes were detected in the different donors, which suggest that some loci for grain size were conserved in Genus *Oryza*. Some loci controlled the opposite phenotype, long grain vs. short grain, wide grain vs. thin grain, validating these loci’s functions in forward and reverse direction and also suggesting that the loci functioned divergence in the different donors. Taken together, these results would provide the information that the loci for grain size from the different donors were the same or different haplotypes; it also indicated that agronomic IL library with the donor of 7 AA genome species was an excellent resource and tool to discover favorable allelic variations and new QTLs or genes for rice improvement.

## Discussion

### Agronomic Introgression Line Library for All AA Genome Species Is an Important Stock for Breeding Improvement in Rice

The geographical distribution of wild relative species with the AA genome is in a wide range of environments, *O. nivara* and *O. rufipogon* mainly in Asia, *O. glaberrima*, *O. barthii*, and *O. longistaminata* in Africa, *O. meridionalis* in Australia, *O. glumaepatula* in Latin America, and wild relative species evolve a large extend on genetic differentiation and morphological variation under the different ecological environments (Vaughan et al., 2005). Constructing introgression lines are a feasible and effective approach to transferring the favorable genes from wild relative species to the cultivated varieties for improving elite cultivars. By now, more than 40 sets of ILs were developed using the AA genome wild species as the donor parents (Chen et al., 2006; Tian et al., 2006; McCouch et al., 2007; Rangel et al., 2008; Hao et al., 2009; Ali et al., 2010; Gutierrez et al., 2010; Ramos et al., 2016; Bhatia et al., 2017), but ILs were almost derived from a single accession of AA genome species in a single background, which leads to the lack of systematic utilization of favorable genes. One of the challenges of constructing interspecific introgression lines was to overcome interspecific hybrid sterility. In this study, we selected the typical 330 accessions of AA genome species distributed in the different geographical region as the donor parents to raise agronomic IL library. We observed that pollen fertility of F_1_ varied from 1.92% to 93.19% dependent on the different accessions of *O. nivara* and *O. rufipogon*, whereas all the crosses with the accessions of *O. barthii*, *O. glumaepatula*, and *O. meridionalis* showed complete pollen sterility in the F_1_ combinations (data not shown). When the *japonica* varieties Dianjingyou 1 and Yundao 1 used as the recurrent parents were crossed with the accession of *O. longistaminata*, the crossing was failed despite many efforts. Only the cross using an *indica* variety RD23 as the recurrent parent and *O. longistaminata* as the donor was obtained by embryo rescue. Fortunately, the female gametes from the interspecific hybrids were partially fertile, and some hybridization seeds in the different combinations could be harvested by backcrossing F_1_ as the female parent with *O. sativa* as the male parent. Finally, agronomic IL library that contains 6,372 lines was developed based on the agronomic trait selection, and agronomic ILs showed a significant difference from the recurrent parents, which include spreading panicle, erect panicle, dense panicle, lax panicle, awn, prostrate growth, plant height, pericarp color, kernel color, glabrous hull, grain size, 1,000-grain weight, drought resistance and aerobic adaption, and blast resistance (Figure 1), and this IL library help us to understand the genetic base of agronomic traits and explore the favorable genes or allelic variations, and also breeding improvement systematically and comprehensively.

Variation for agronomic traits in this IL library shows the importance of AA genome species for further breeding improvement in rice. For example, 18 ILs in Dianjingyou 1 background and 57 ILs in Yundao 1 background showed dense panicle (Supplementary Tables 2–4), 137 ILs registered significant improvement over the recurrent parents in grain weight (data not shown), and these ILs derived from multiple donors could contribute to the variations for yield.

The upland rice is a predominant ecotype adapted to aerobic and rain-fed conditions in the mountainous areas that have high genetic variability in the characteristics of morphology and physiology, such as glabrous hull and aerobic adaptation (Bridhikitti and Overcamp, 2012; Sandhu et al., 2013). In this study, 160 accessions of upland rice that represent an abundant genetic diversity were used to raise introgression lines. Among those agronomic ILs, 93 and 18 ILs in the Diangjingyou 1 and Yundao 1 background exhibited the glabrous hull phenotype. Those resources were not only good for breeding varieties suitable for agricultural operation, but also helpful to understand the genetic mechanism of glabrous hull development. A total of 125 ILs in aerobic adaptation were superior to the recurrent parents, which are important and environmental-friendly breeding materials to meet the need of aerobic rice development.

The effect of alleles on the agronomic traits varied with genetic background. The different recurrent parents helped us to find background-dependent useful traits or stable traits in the different backgrounds. In this study, with the donor accessions of *O. barthii*, *O. glumaepatula*, *O. meridionalis*, *O. nivara*, *O. rufipogon*, and upland rice of *O. sativa*, 62, 37, 147, 470, 122, and 710 ILs that conferred agronomic traits were found in the genetic background of the Dianjingyou 1 and Yundao 1, which suggested that the genes for agronomic traits had a stable effect on the different genetic backgrounds (Supplementary Tables 2–4). In addition, we found ILs that showed aerobic adaptation with the donors of *O. barthii*, *O. glumaepatula*, *O. meridionalis*, *O. nivara*, *O. rufipogon* were detected in the Yundao 1 background, rather than Dianjingyou 1 background (Supplementary Tables 2–4). The results would provide the theoretical guidance of the relationship between the traits and genetic background in rice breeding.

### Exploration of Natural Allelic Variations Using Agronomic Introgression Line Library

Genetic diversity and allele were lost during the domestication from the wild species of rice to the cultivated rice (Sun et al., 2002), whereas narrow genetic basis led to the yield bottleneck of Asian cultivated rice. In the recent years, mining and utilization of useful allele variation have made great progress in rice breeding. For example, the allelic variation in the *Wx* gene and *SSSI* was proved to contribute greatly to the differences in rice eating and cooking qualities (ECQs) in the two subspecies (Li et al., 2018). Allelic variation at the *E1/Ghd7* locus allowed an expansion of the rice cultivation area through adjusting heading date (Saito et al., 2019). The allele types of *BPH9* conferred varying levels of resistance to different biotypes of *BPH* and enabled rice to combat planthopper variation (Zhao et al., 2016). The allelic variation at the rice blast resistance (*R*) *Pid3* locus was analyzed based on the 3K RGP sequencing data, and different strategies were developed to apply the functional *Pid3* alleles to *indica* and *japonica* rice breeding (Lv et al., 2017). In this study, one locus for GL and one locus for GW were explored from the six and five different donor species, respectively. Two loci for GL, three loci for GW, and one locus for the RLW were detected from the donors of four species, respectively (Figures 3–5). Additionally, many of published genes for grain size were found based on the agronomic introgression library analysis, such as *GW2* (Song et al., 2007), *GL2* (Hu et al., 2015), *PGL2* (Heang and Sassa, 2012b), *PGL1* (Heang and Sassa, 2012a), *GL3.2/CYP78A13* (Xu F. et al., 2015), *GS3* (Fan et al., 2006; Takano-Kai et al., 2009;Mao et al., 2010), *qGL3-1* (Qi et al., 2012), *qGL3.3* (Hu et al., 2018), *GS6* (Sun et al., 2013), *TGW6* (Ishimaru et al., 2013), *GL7* (Wang et al., 2015), and *OsSPL13* (Si et al., 2016). In addition, 22 loci might be new QTLs or genes controlled for GL and GW from the different AA genome donors. Accordingly, agronomic introgression library with multiple donors from different relatives of Asian cultivated rice is a powerful resource platform to discover novel and functional allelic variations for agronomic traits. Mining the natural functional variations in the useful genes derived from the multiple donors and combing the different alleles through diversification could be useful for an accurate rice breeding program.

Therefore, agronomic IL libraries derived from the multiple donors have some advantages: (1) an abundant genetic variations were introgressed into the cultivated rice genome; (2) target genes or QTLs for the same phenotype could be validated by the different donors, and it will provide the information that these target genes or QTLs could be the same haplotype; (3) the genes or QTLs responsible for the opposite phenotypes, for example, long-grain size and short-grain size, could also be confirmed using the different populations from multiple donors, and it could be the different haplotypes. Therefore, this agronomic IL library will help us to improve rice breeding and interesting gene discovery and utilization.

## Data Availability Statement

The datasets presented in this study can be found in online repositories. The names of the repository/repositories and accession number(s) can be found in the article/Supplementary Material.

## Author Contributions

YZ and DT draft the manuscript. DT designed the research. JZ, PX, WD, and XD developed the introgression lines. YZ, YYa, YYu, JL, and QP participated the genotype and phenotype evaluation. YZ performed the data analysis. All authors reviewed and approved the final manuscript.

## Conflict of Interest

The authors declare that the research was conducted in the absence of any commercial or financial relationships that could be construed as a potential conflict of interest.

## Publisher’s Note

All claims expressed in this article are solely those of the authors and do not necessarily represent those of their affiliated organizations, or those of the publisher, the editors and the reviewers. Any product that may be evaluated in this article, or claim that may be made by its manufacturer, is not guaranteed or endorsed by the publisher.

## Footnotes

1
http://www.gramene.org

## Supplementary Material

Supplementary Figure 1 Physical map of the 168 SSR markers in rice used for this study.

Supplementary Figure 2 Chromosome segments distribution and coverage degree of introgression library from the donor of *O. barthii*. Each block at the row represented introgression from a total of 29 ILs on the target chromosome, regardless of introgression segments in other chromosomes, and each column represented a molecular marker locus.

Supplementary Figure 3 Chromosome segments distribution and coverage degree of introgression library from the donor of *O. glumaepatula*. Each block at the row represented introgression from a total of 30 ILs on the target chromosome, regardless of introgression segments in other chromosomes, and each column represented a molecular marker locus.

Supplementary Figure 4 Chromosome segments distribution and coverage degree of introgression library from the donor of *O. meridionalis*. Each block at the row represented introgression from a total of 76 ILs on the target chromosome, regardless of introgression segments in other chromosomes, and each column represented a molecular marker locus.

Supplementary Figure 5 Chromosome segments distribution and coverage degree of introgression library from the donor of *O. nivara*. Each block at the row represented introgression from a total of 380 ILs on the target chromosome, regardless of introgression segments in other chromosomes, and each column represented a molecular marker locus.

Supplementary Figure 6 Chromosome segments distribution and coverage degree of introgression library from the donor of *O. rufipogon*. Each block at the row represented introgression from a total of 74 ILs on the target chromosome, regardless of introgression segments in other chromosomes, and each column represented a molecular marker locus.

Supplementary Figure 7 Chromosome segments distribution and coverage degree of introgression library from the donor of *O. glaberrima.* Each block at the row represented introgression from a total of 81 ILs on the target chromosome, regardless of introgression segments in other chromosomes, and each column represented a molecular marker locus.

Supplementary Figure 8 Chromosome segments distribution and coverage degree of introgression library from the donor of upland rice. Each block at the row represented introgression from a total of 731 ILs on the target chromosome, regardless of introgression segments in other chromosomes, and each column represented a molecular marker locus.
