# Supplementary material for: A Genetic Resource for Rice Improvement: Introgression Library of Agronomic Traits for All AA Genome Oryza Species
Source: Front Plant Sci. 2022 Mar 24;13:856514. doi: 10.3389/fpls.2022.856514 (PMC8992386; doi:10.3389/fpls.2022.856514)
Supplement: Supplementary file 3 [file Data_Sheet_1.docx]

Supplementary Table 2. Evaluation summary of introgression lines (ILs) from the AA genome wild relatives of rice and upland rice in Dianjingyou 1 background

| Trait | *O. barthii* | | | *O. glumaepatula* | | | *O. meridionalis* | | | *O. nivara* | | | *O. rufipogon* | | | Upland rice | | | | Total |
| --- | --- | --- | --- | --- | --- | --- | --- | --- | --- | --- | --- | --- | --- | --- | --- | --- | --- | --- | --- | --- |
|  | No. of the donors | No. of ILs | Generation | No. of the donors | No. of ILs | Generation | No.of the donors | No. of ILs | Generation | No. of the donors | No. of ILs | Generation | No. of the donors | No. of ILs | Generation | No. of the donors | | No. of ILs | Generation |  |
| Spreading panicle | 4 | 16 | BC_3_F_7_ | 1 | 5 | BC_3_F_7_ | 3 | 28 | BC_3_F_7_ | 12 | 109 | BC_2_-BC_3_ | 3 | 16 | BC_2_-BC_3_ | 46 | 103 | | BC_2_F_8_ | 277 |
| Erect panicle | 2 | 2 | BC_3_F_7_ | 1 | 1 | BC_3_F_7_ | 2 | 4 | BC_3_F_7_ | 2 | 4 | BC_2_-BC_3_ |  |  | BC_3_F_7_ | 5 | 10 | | BC_2_F_8_ | 21 |
| Dense panicle |  |  |  |  |  |  |  |  |  | 1 | 1 | BC_2_-BC_3_ |  |  |  | 5 | 13 | | BC_2_F_8_ | 14 |
| Lax panicle |  |  |  |  |  |  |  |  |  | 5 | 10 | BC_2_-BC_3_ | 2 | 2 | BC_2_-BC_3_ | 2 | 2 | | BC_2_F_8_ | 14 |
| Awn | 2 | 5 | BC_3_F_7_ | 2 | 4 | BC_3_F_7_ | 4 | 10 | BC_3_F_7_ | 11 | 41 | BC_2_-BC_3_ | 2 | 4 | BC_2_-BC_3_ | 6 | 9 | | BC_2_F_8_ | 73 |
| Prostrate growth | 1 | 3 | BC_3_F_7_ |  |  | BC_3_F_7_ | 1 | 5 | BC_3_F_7_ | 6 | 19 | BC_2_-BC_3_ |  |  |  | 2 | 2 | | BC_2_F_8_ | 29 |
| Plant height | 2 | 2 | BC_3_F_7_ | 2 | 2 | BC_3_F_7_ | 3 | 5 | BC_3_F_7_ | 16 | 53 | BC_2_-BC_3_ | 6 | 10 | BC_3_F_7_ | 33 | 50 | | BC_2_F_8_ | 123 |
| Pericarp color | 2 | 2 | BC_3_F_7_ |  |  |  |  |  |  | 6 | 12 | BC_2_-BC_3_ | 1 | 1 | BC_2_-BC_3_ | 4 | 4 | | BC_2_F_8_ | 19 |
| Kernel color |  |  |  |  |  |  | 1 | 1 | BC_3_F_7_ | 6 | 15 | BC_2_-BC_3_ |  |  |  | 9 | 16 | | BC_2_F_8_ | 32 |
| Glabrous hull |  |  |  |  |  |  |  |  |  |  |  | BC_2_-BC_3_ |  |  |  | 20 | 93 | | BC_2_F_8_ | 93 |
| Grain length | 4 | 19 | BC_3_F_7_ | 2 | 15 | BC_3_F_7_ | 10 | 37 | BC_3_F_7_ | 24 | 204 | BC_2_-BC_3_ | 10 | 44 | BC_2_-BC_3_ | 62 | 368 | | BC_2_F_8_ | 687 |
| Grain width | 4 | 7 | BC_3_F_7_ | 4 | 12 | BC_2_-BC_3_ | 7 | 17 | BC_3_F_7_ | 22 | 114 | BC_2_-BC_3_ | 8 | 26 | BC_2_-BC_3_ | 49 | 113 | | BC_2_F_8_ | 289 |
| 1,000-grain weight | 7 | 18 | BC_3_F_7_ | 4 | 22 | BC_3_F_7_ | 9 | 72 | BC_3_F_7_ | 26 | 242 | BC_2_-BC_3_ | 9 | 32 | BC_2_-BC_3_ | 125 | 695 | | BC_2_F_8_ | 1081 |
| Drought-resistance |  |  |  |  |  |  |  |  |  |  |  |  |  |  | BC3F7 | 32 | 35 | | BC_2_F_8_ | 35 |
| Aerobic adaption |  |  |  |  |  |  |  |  |  |  |  |  |  |  | BC3F7 | 32 | 48 | | BC_2_F_8_ | 48 |
| Total |  | 74 |  |  | 61 |  |  | 179 |  |  | 824 |  |  | 135 |  |  | 1561 | |  | 2834 |

Supplementary Table 3. Evaluation summary of introgression lines from *O. glaberrima* in Dianjingyou 1 background

| Trait | No. of Donors | No. of ILs | Generation | Total |
| --- | --- | --- | --- | --- |
|  |  |  |  |  |
| Spreading panicle | 7 | 15 | BC_4_F_9_ | 17 |
|  | 2 | 2 | BC_6_F_7_ |  |
| Erect panicle | 1 | 1 | BC_3_F_10_ | 1 |
| Dense panicle | 2 | 4 | BC_4_F_9_ | 4 |
| Lax panicle | 2 | 8 | BC_4_F_9_ | 8 |
| Panicle length | 3 | 6 | BC_4_F_9_ | 18 |
|  | 1 | 3 | BC_2_F_11_ |  |
|  | 2 | 4 | BC_4_F_9_ |  |
|  | 2 | 5 | BC_5_F_8_ |  |
| Awn | 1 | 4 | BC_2_F_11_ | 4 |
| Prostrate growth | 1 | 2 | BC_2_F_11_ | 2 |
| Plant height | 1 | 1 | BC_4_F_9_ | 10 |
|  | 1 | 3 | BC_5_F_8_ |  |
|  | 1 | 6 | BC_2_F_11_ |  |
| Tiller number | 2 | 4 | BC_4_F_9_ | 6 |
|  | 1 | 2 | BC_5_F_8_ |  |
| Pericarp color | 1 | 1 | BC_4_F_11_ | 1 |
| Glabrous hull | 1 | 2 | BC_3_F_10_ | 8 |
|  | 1 | 6 | BC_4_F_9_ |  |
| Long empty glume | 1 | 2 | BC_3_F_10_ | 6 |
|  | 1 | 2 | BC_4_F_9_ |  |
|  | 1 | 2 | BC_6_F_7_ |  |
| Grain length | 2 | 2 | BC_2_F_17_ | 99 |
|  | 1 | 2 | BC_4_F_9_ |  |
|  | 2 | 5 | BC_5_F_8_ |  |
|  | 1 | 2 | BC_6_F_7_ |  |
|  | 1 | 12 | BC_2_F_11_ |  |
|  | 5 | 32 | BC_3_F_10_ |  |
|  | 8 | 35 | BC_4_F_9_ |  |
|  | 3 | 9 | BC_6_F_7_ |  |
| Grain width | 2 | 2 | BC_4_F_9_ | 5 |
|  | 1 | 3 | BC_5_F_8_ |  |
| 1,000-Grain weight | 1 | 2 | BC_2_F_11_ | 57 |
|  | 4 | 18 | BC_3_F_10_ |  |
|  | 4 | 13 | BC_4_F_9_ |  |
|  | 3 | 7 | BC_5_F_8_ |  |
|  | 1 | 2 | BC_2_F_11_ |  |
|  | 2 | 5 | BC_3_F_10_ |  |
|  | 3 | 8 | BC_4_F_9_ |  |
|  | 1 | 2 | BC_5_F_8_ |  |
| Seed shattering | 1 | 2 | BC_3_F_10_ | 2 |
| Senescence | 1 | 1 | BC_5_F_8_ | 1 |
| Total |  |  |  | 251 |

Supplementary Table 4. Evaluation summary of introgression lines from the AA genome wild relatives of rice and upland rice in Yundao 1 background

| Traits | *O. barthii* | | *O. glumaepatula* | | *O. meridionalis* | | *O. nivara* | | *O. rufipogon* | | Upland rice | | Total |
| --- | --- | --- | --- | --- | --- | --- | --- | --- | --- | --- | --- | --- | --- |
|  | No. of the donors | No. of ILs | No. of the donors | No. of ILs | No. of the donors | No. of ILs | No. of the donors | No. of ILs | No. of the donors | No. of ILs | No .of the donors | No. of ILs |  |
| Spreading panicle | 3 | 6 | 1 | 6 | 4 | 14 | 4 | 22 | 6 | 21 | 13 | 25 | 94 |
| Erect panicle | 3 | 11 |  |  | 6 | 54 | 4 | 15 | 8 | 29 | 2 | 5 | 114 |
| Dense panicle |  |  |  |  |  |  | 1 | 2 |  |  | 12 | 55 | 57 |
| Lax panicle | 1 | 1 |  |  | 1 | 1 | 8 | 11 | 8 | 12 | 16 | 35 | 60 |
| Awn | 5 | 15 | 2 | 2 | 7 | 14 | 8 | 21 | 12 | 44 | 2 | 2 | 98 |
| Prostrate growth | 2 | 3 |  |  | 2 | 4 | 6 | 15 | 3 | 4 | 1 | 1 | 27 |
| Plant height | 4 | 12 | 2 | 6 | 9 | 25 | 14 | 52 | 19 | 60 | 13 | 27 | 182 |
| Pericarp color |  |  |  |  | 2 | 4 | 4 | 8 | 6 | 11 | 12 | 13 | 36 |
| Kernel color | 1 | 1 |  |  | 5 | 9 | 2 | 4 | 4 | 13 | 2 | 2 | 29 |
| Glabrous hull |  |  |  |  | 2 | 2 | 2 | 5 | 2 | 2 | 7 | 18 | 27 |
| Grain length | 6 | 27 | 2 | 6 | 5 | 62 | 10 | 91 | 17 | 111 | 20 | 67 | 364 |
| Grain width | 2 | 12 |  |  |  |  |  |  | 7 | 13 | 1 | 1 | 26 |
| 1,000-grain weight | 8 | 119 | 8 | 54 | 13 | 331 | 21 | 448 | 26 | 506 | 83 | 460 | 1918 |
| Drought-resistance | 3 | 3 |  |  | 1 | 6 | 1 | 8 | 2 | 7 | 34 | 67 | 91 |
| Aerobic adaption | 5 | 34 | 3 | 11 | 6 | 21 | 8 | 12 | 10 | 25 | 27 | 47 | 150 |
| Total |  | 244 |  | 85 |  | 547 |  | 714 |  | 858 |  | 825 | 3273 |

Note: BC_3_F_7_ ILs derived from the wild relatives of rice as the donor were developed，and BC_2_F_8_ ILs derived from the upland rice as the donor were built.

Supplementary Table 5. Evaluation summary of introgression lines from *O. longistaminata* in RD23 background

| Traits | No.of ILs | Generations | Total |
| --- | --- | --- | --- |
| Plant height | 4 | BC_2_F_10_ | 10 |
|  | 6 | BC_3_F_9_ |  |
| Awn | 4 | BC_2_F_10_ | 17 |
|  | 8 | BC_3_F_9_ |  |
|  | 5 | BC_4_F_8_ |  |
| Kernel color | 1 | BC_3_F_14_ | 2 |
|  | 1 | BC_4_F_9_ |  |
| Grain length | 14 | BC_2_F_10_ | 25 |
|  | 6 | BC_3_F_9_ |  |
|  | 5 | BC_4_F_8_ |  |
| Grain width | 14 | BC_2_F_10_ | 81 |
|  | 41 | BC_3_F_9_ |  |
|  | 22 | BC_4_F_8_ |  |
|  | 4 | BC_5_F_11_ |  |
| 1,000-grain weight | 45 | BC_2_F_10_ | 126 |
|  | 17 | BC_3_F_9_ |  |
|  | 43 | BC_4_F_8_ |  |
|  | 6 | BC_1_F_21_ |  |
|  | 15 | BC_5_F_11_ |  |
| Blast-resistance | 4 | BC_3_F_14_ | 4 |
| Total |  |  | 265 |

Supplementary Table 7. Chromosome coverage of substituted segments in introgression lines from the donor of *O. barthii*

| Chr | Length | Times | Coverage length | Coverage rate (%) |
| --- | --- | --- | --- | --- |
| 1 | 129.64 | 2.99 | 37.40 | 86.44 |
| 2 | 75.14 | 2.09 | 25.45 | 70.82 |
| 3 | 176.11 | 4.83 | 36.41 | 100.00 |
| 4 | 110.55 | 3.11 | 27.06 | 76.22 |
| 5 | 72.67 | 2.42 | 25.16 | 83.98 |
| 6 | 83.48 | 2.67 | 25.61 | 81.95 |
| 7 | 85.04 | 2.86 | 27.34 | 92.06 |
| 8 | 113.56 | 3.99 | 28.44 | 100.00 |
| 9 | 84.59 | 3.67 | 23.01 | 100.00 |
| 10 | 29.31 | 1.26 | 19.83 | 85.45 |
| 11 | 34.84 | 1.20 | 11.36 | 39.14 |
| 12 | 46.50 | 1.68 | 15.98 | 58.04 |
| Genome | 1041.42 | 2.79 | 303.07 | 81.19 |

Supplementary Table 8. Chromosome coverage of substituted segments in introgression lines from the donor of *O. glumaepatula*

| Chr | Length | Times | Coverage length | Coverage rate (%) |
| --- | --- | --- | --- | --- |
| 1 | 89.97 | 2.07 | 30.92 | 71.45 |
| 2 | 49.25 | 1.37 | 24.10 | 67.05 |
| 3 | 289.86 | 7.96 | 36.41 | 100.00 |
| 4 | 60.00 | 1.68 | 26.84 | 75.58 |
| 5 | 51.27 | 1.71 | 14.27 | 47.62 |
| 6 | 75.55 | 2.41 | 28.62 | 91.60 |
| 7 | 28.18 | 0.94 | 17.93 | 60.37 |
| 8 | 18.63 | 0.65 | 15.83 | 55.66 |
| 9 | 81.52 | 3.54 | 21.40 | 92.99 |
| 10 | 41.76 | 1.79 | 22.23 | 95.80 |
| 11 | 32.07 | 1.10 | 17.47 | 60.19 |
| 12 | 25.58 | 0.92 | 16.88 | 61.31 |
| Genome | 843.65 | 2.26 | 272.91 | 73.11 |

Supplementary Table 9. Chromosome coverage of substituted segments in introgression lines from the donor of *O. meridionalis*

| Chr | Length | Times | Coverage length | Coverage rate (%) |
| --- | --- | --- | --- | --- |
| 1 | 96.76 | 2.23 | 42.30 | 97.76 |
| 2 | 138.03 | 3.84 | 29.47 | 81.99 |
| 3 | 244.21 | 6.70 | 36.41 | 100.00 |
| 4 | 225.45 | 6.35 | 32.78 | 92.33 |
| 5 | 143.85 | 4.80 | 24.27 | 81.00 |
| 6 | 90.33 | 2.89 | 31.25 | 100.00 |
| 7 | 65.94 | 2.22 | 24.18 | 81.40 |
| 8 | 142.03 | 4.99 | 28.44 | 100.00 |
| 9 | 143.41 | 6.23 | 21.65 | 94.09 |
| 10 | 74.06 | 3.19 | 21.05 | 90.69 |
| 11 | 104.86 | 3.61 | 17.62 | 60.71 |
| 12 | 132.92 | 4.82 | 23.43 | 85.10 |
| Genome | 1601.86 | 4.29 | 332.86 | 89.17 |

Supplementary Table 10. Chromosome coverage of substituted segments in introgression lines from the donor of *O. nivara*

| Chr | Length | Times | Coverage length | Coverage rate (%) |
| --- | --- | --- | --- | --- |
| 1 | 1200.24 | 27.73 | 43.27 | 100.00 |
| 2 | 996.39 | 27.72 | 31.16 | 86.70 |
| 3 | 1949.39 | 53.53 | 36.41 | 100.00 |
| 4 | 1045.16 | 29.43 | 35.50 | 100.00 |
| 5 | 905.14 | 30.21 | 28.66 | 95.65 |
| 6 | 1329.89 | 42.55 | 31.25 | 100.00 |
| 7 | 456.48 | 15.37 | 26.80 | 90.25 |
| 8 | 858.69 | 30.18 | 28.44 | 100.00 |
| 9 | 1062.76 | 46.18 | 23.01 | 100.00 |
| 10 | 459.32 | 19.79 | 23.21 | 100.00 |
| 11 | 590.07 | 20.33 | 29.02 | 100.00 |
| 12 | 889.97 | 32.32 | 25.95 | 94.26 |
| Genome | 11743.50 | 31.46 | 362.69 | 97.17 |

Supplementary Table 11. Chromosome coverage of substituted segments in introgression lines from the donor of *O. rufipogon*

| Chr | Length | Times | Coverage length | Coverage rate (%) |
| --- | --- | --- | --- | --- |
| 1 | 266.09 | 6.14 | 39.54 | 91.36 |
| 2 | 241.52 | 6.72 | 26.75 | 74.43 |
| 3 | 462.33 | 12.69 | 36.41 | 100.00 |
| 4 | 142.56 | 4.01 | 29.68 | 83.59 |
| 5 | 147.03 | 4.90 | 23.79 | 79.40 |
| 6 | 168.65 | 5.39 | 31.25 | 100.00 |
| 7 | 154.01 | 5.18 | 28.37 | 95.52 |
| 8 | 162.50 | 5.71 | 28.44 | 100.00 |
| 9 | 163.18 | 7.09 | 23.01 | 100.00 |
| 10 | 105.36 | 4.53 | 20.81 | 89.65 |
| 11 | 170.07 | 5.86 | 23.44 | 80.77 |
| 12 | 191.41 | 6.95 | 21.43 | 77.84 |
| Genome | 2374.71 | 6.36 | 332.92 | 89.19 |

Supplementary Table 12. Chromosome coverage of substituted segments in introgression lines from the donor of *O. glaberrima*

| Chr | Length | Times | Coverage length | Coverage rate (%) |
| --- | --- | --- | --- | --- |
| 1 | 370.47 | 8.56 | 35.23 | 81.41 |
| 2 | 344.50 | 9.58 | 33.37 | 92.86 |
| 3 | 586.15 | 16.09 | 36.41 | 100.00 |
| 4 | 258.15 | 7.27 | 30.13 | 84.87 |
| 5 | 311.56 | 10.39 | 28.59 | 95.41 |
| 6 | 405.41 | 12.97 | 31.25 | 100.00 |
| 7 | 314.81 | 10.60 | 25.48 | 85.78 |
| 8 | 254.34 | 8.94 | 24.29 | 85.38 |
| 9 | 249.29 | 10.83 | 23.01 | 100.00 |
| 10 | 204.82 | 8.82 | 18.25 | 78.65 |
| 11 | 403.49 | 13.90 | 29.02 | 100.00 |
| 12 | 148.42 | 5.39 | 23.53 | 85.46 |
| Genome | 3851.42 | 10.31 | 338.56 | 90.70 |

Supplementary Table 13. Chromosome coverage of substituted segments in introgression lines from the donor of upland rice in *O. sativa*

| Chr | Length | Times | Coverage length | Coverage rate (%) |
| --- | --- | --- | --- | --- |
| 1 | 5311.73 | 122.75 | 43.27 | 100.00 |
| 2 | 2822.51 | 78.53 | 35.94 | 100.00 |
| 3 | 4055.68 | 111.37 | 36.41 | 100.00 |
| 4 | 2950.88 | 83.11 | 35.50 | 100.00 |
| 5 | 3291.60 | 109.87 | 29.96 | 100.00 |
| 6 | 3387.60 | 108.40 | 31.25 | 100.00 |
| 7 | 2412.21 | 81.22 | 29.70 | 100.00 |
| 8 | 2639.73 | 92.80 | 28.44 | 100.00 |
| 9 | 3139.13 | 136.40 | 23.01 | 100.00 |
| 10 | 982.65 | 42.34 | 19.85 | 85.53 |
| 11 | 3317.60 | 114.31 | 29.02 | 100.00 |
| 12 | 1794.66 | 65.18 | 27.53 | 100.00 |
| Genome | 36105.97 | 96.73 | 369.89 | 99.10 |

Supplementary Table 14. The correlation of grain size in the different environments

| Traits |  | GL | | GW | | RLW | |
| --- | --- | --- | --- | --- | --- | --- | --- |
|  |  | E1 | E2 | E1 | E2 | E1 | E2 |
| GL | E1 | 1.00** |  |  |  |  |  |
|  | E2 | 0.90** | 1.00** |  |  |  |  |
| GW | E1 | -0.12 | -0.11 | 1.00** |  |  |  |
|  | E2 | -0.06 | -0.08 | 0.78** | 1.00** |  |  |
| RLW | E1 | 0.81** | 0.74** | -0.67** | -0.49** | 1.00** |  |
|  | E2 | 0.71** | 0.79** | -0.55** | -0.66** | 0.85** | 1.00** |

Note:* *P* < 0.05, ** *P* < 0.01
