# Supplementary figures and images for: A Genetic Resource for Rice Improvement: Introgression Library of Agronomic Traits for All AA Genome Oryza Species
Source: Front Plant Sci. 2022 Mar 24;13:856514. doi: 10.3389/fpls.2022.856514 (PMC8992386; doi:10.3389/fpls.2022.856514)

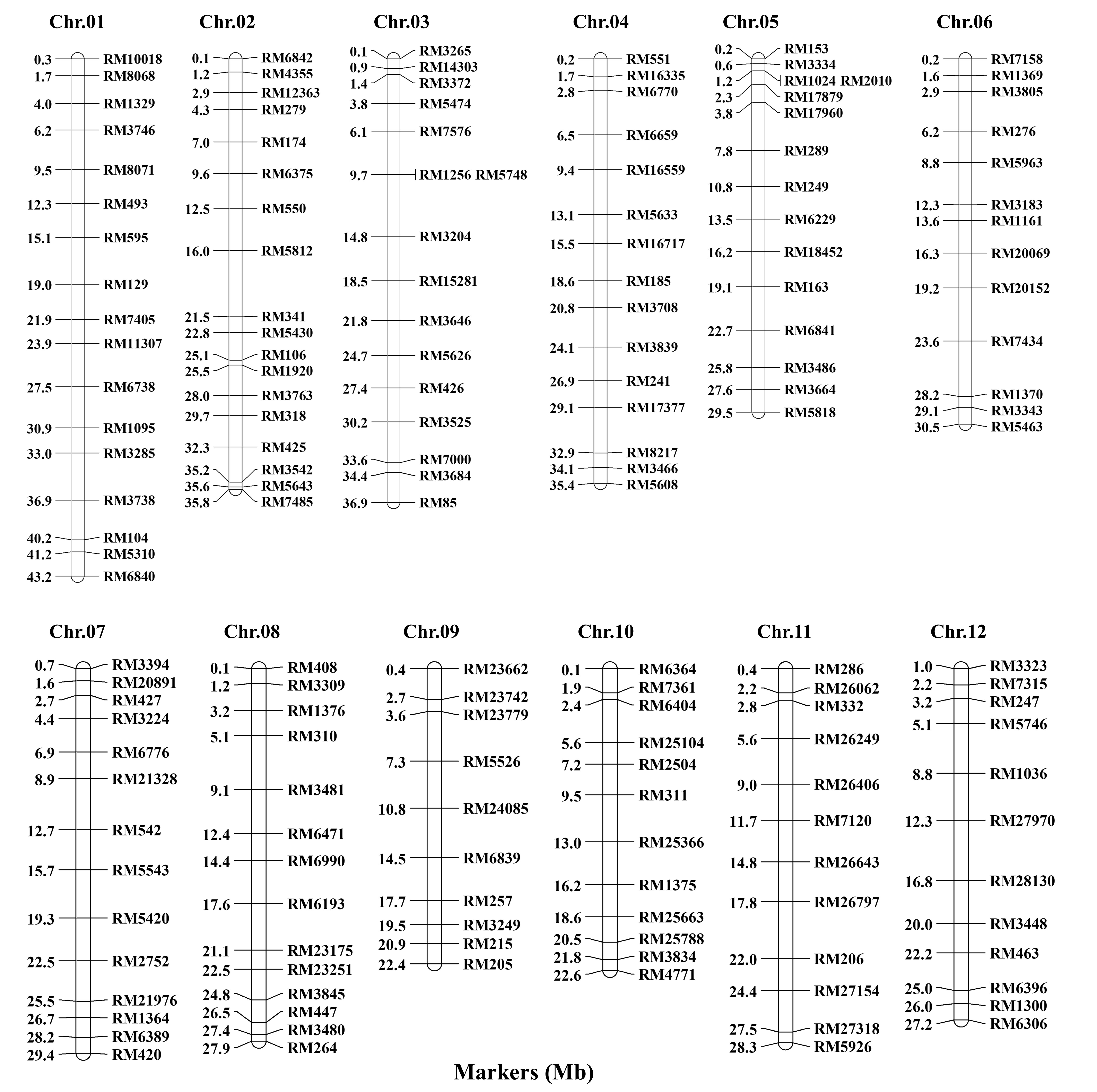

Supplement: Supplementary Figure 1 — Physical map of the 168 SSR markers in rice used for this study. [file Image_1.JPEG]

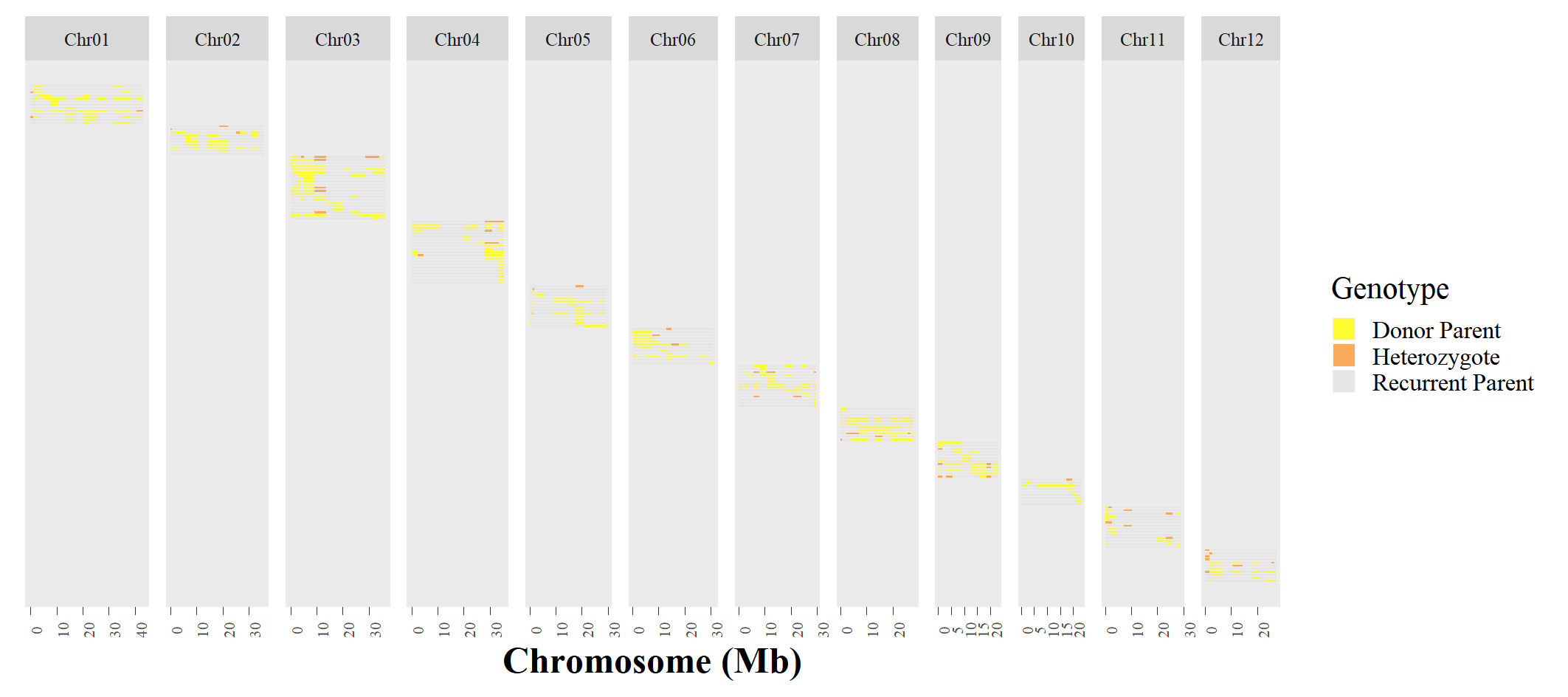

Supplement: Supplementary Figure 2 — Chromosome segments distribution and coverage degree of introgression library from the donor of O. barthii. Each block at the row represented introgression from a total of 29 ILs on the target chromosome, regardless of introgression segments in other chromosomes, and each column represented a molecular marker locus. [file Image_2.TIFF]

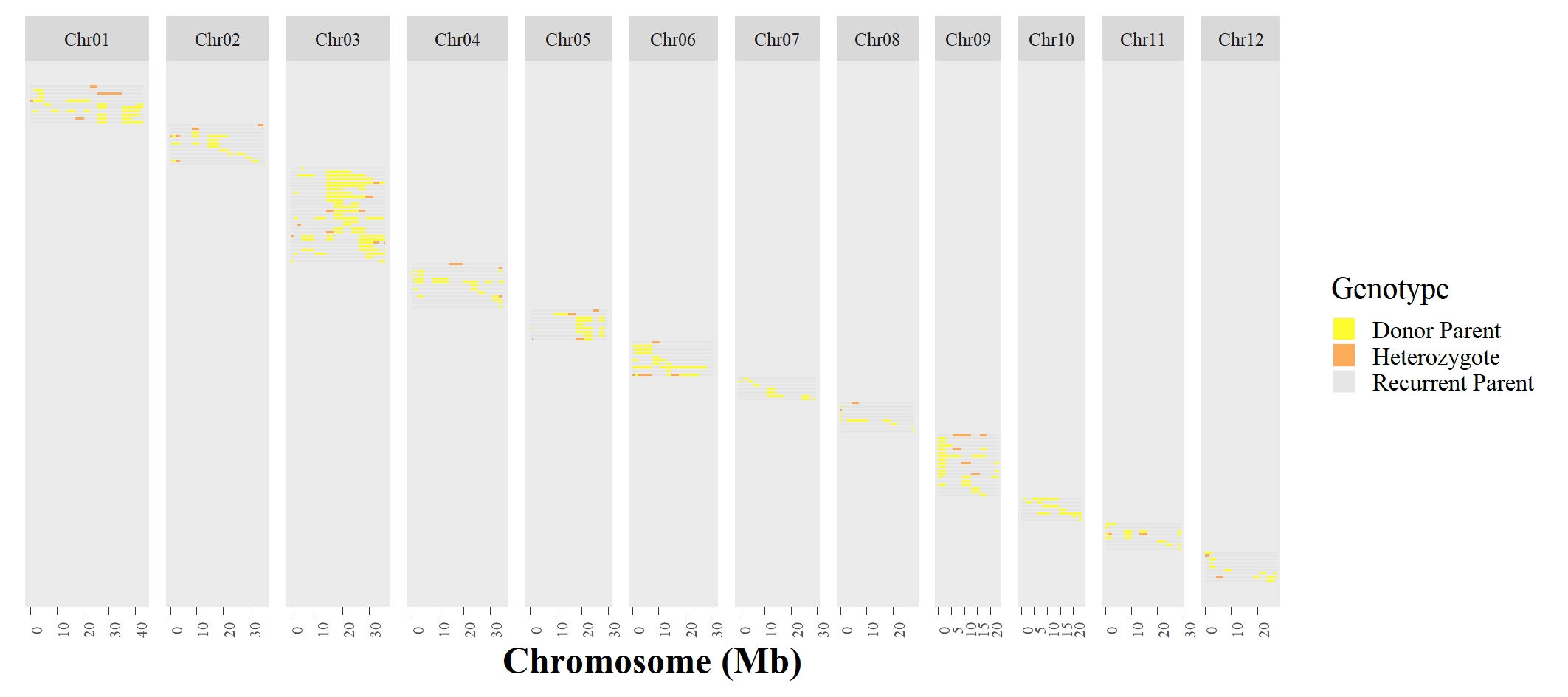

Supplement: Supplementary Figure 3 — Chromosome segments distribution and coverage degree of introgression library from the donor of O. glumaepatula. Each block at the row represented introgression from a total of 30 ILs on the target chromosome, regardless of introgression segments in other chromosomes, and each column represented a molecular marker locus. [file Image_3.TIFF]

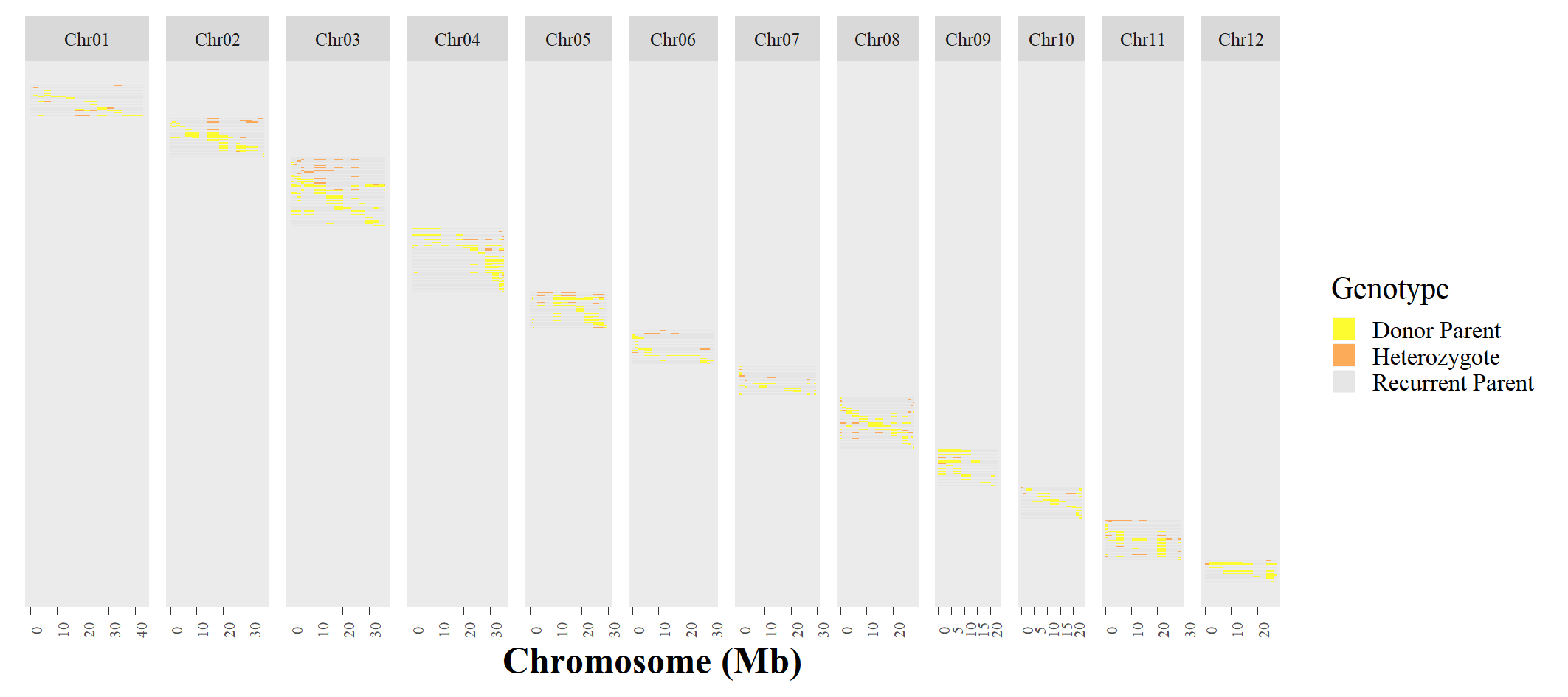

Supplement: Supplementary Figure 4 — Chromosome segments distribution and coverage degree of introgression library from the donor of O. meridionalis. Each block at the row represented introgression from a total of 76 ILs on the target chromosome, regardless of introgression segments in other chromosomes, and each column represented a molecular marker locus. [file Image_4.TIFF]

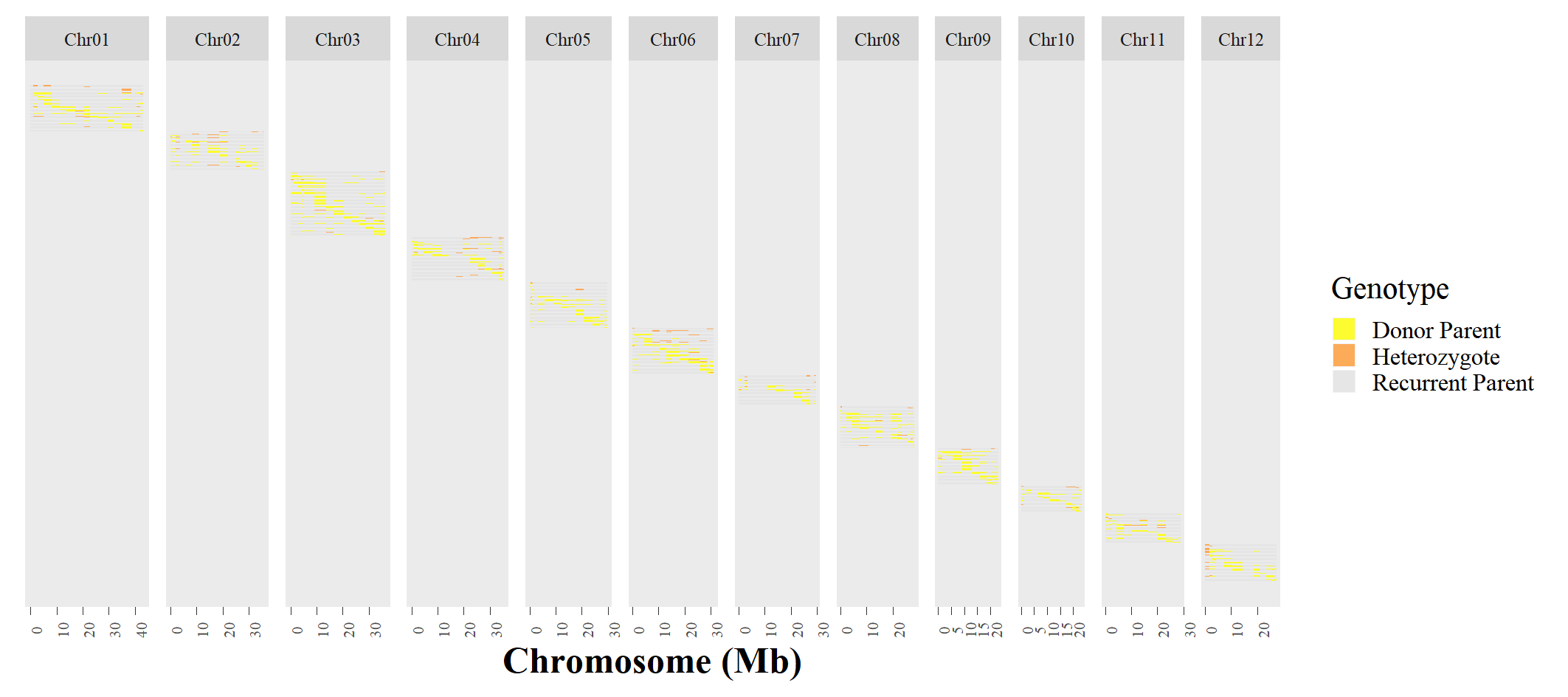

Supplement: Supplementary Figure 5 — Chromosome segments distribution and coverage degree of introgression library from the donor of O. nivara. Each block at the row represented introgression from a total of 380 ILs on the target chromosome, regardless of introgression segments in other chromosomes, and each column represented a molecular marker locus. [file Image_5.TIFF]

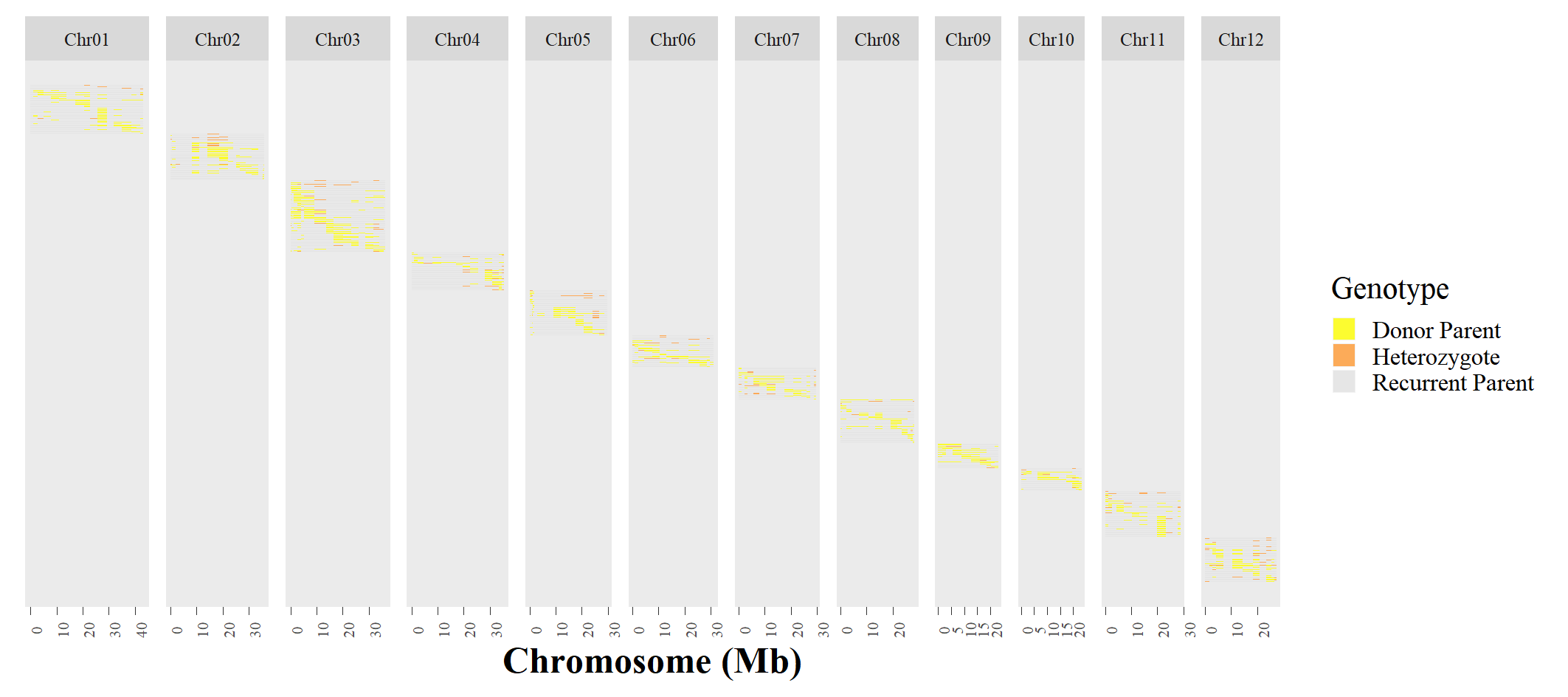

Supplement: Supplementary Figure 6 — Chromosome segments distribution and coverage degree of introgression library from the donor of O. rufipogon. Each block at the row represented introgression from a total of 74 ILs on the target chromosome, regardless of introgression segments in other chromosomes, and each column represented a molecular marker locus. [file Image_6.TIFF]

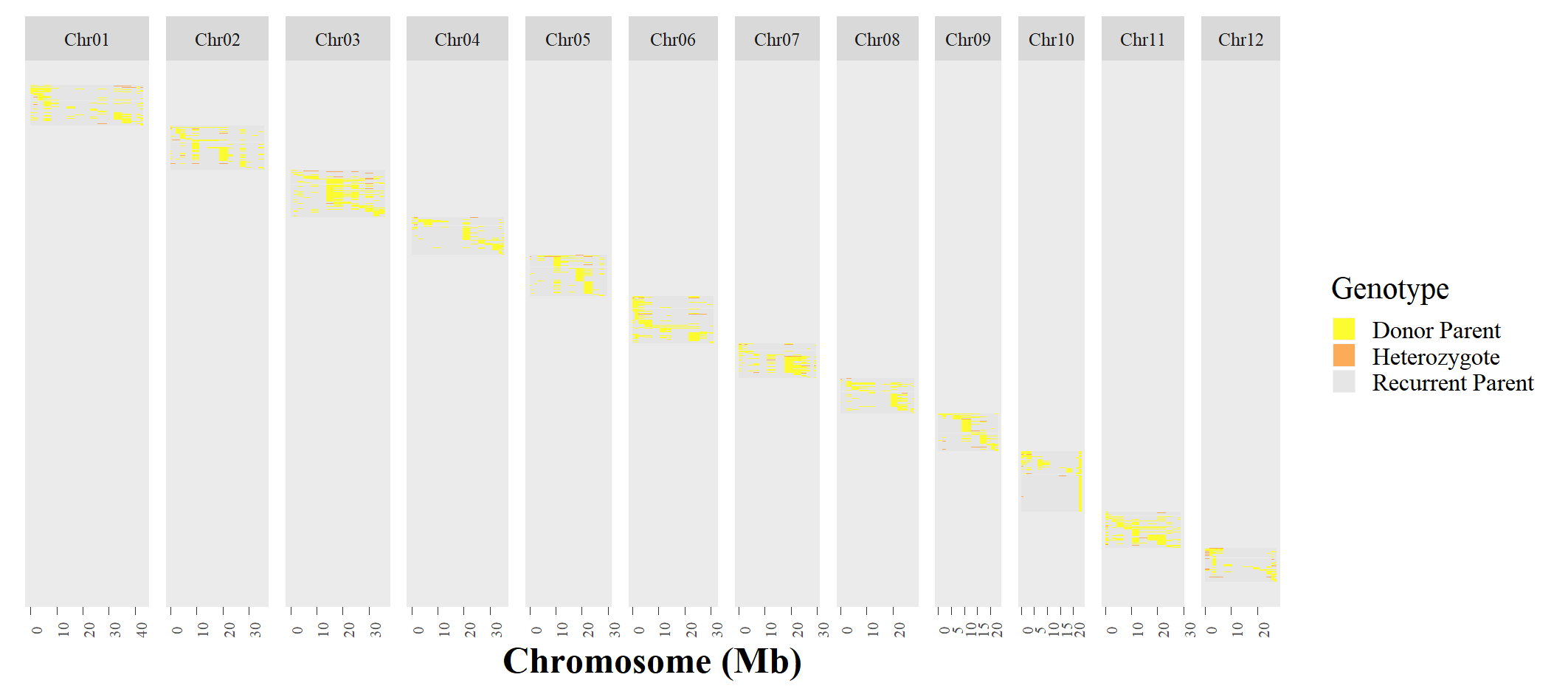

Supplement: Supplementary Figure 7 — Chromosome segments distribution and coverage degree of introgression library from the donor of O. glaberrima. Each block at the row represented introgression from a total of 81 ILs on the target chromosome, regardless of introgression segments in other chromosomes, and each column represented a molecular marker locus. [file Image_7.TIFF]

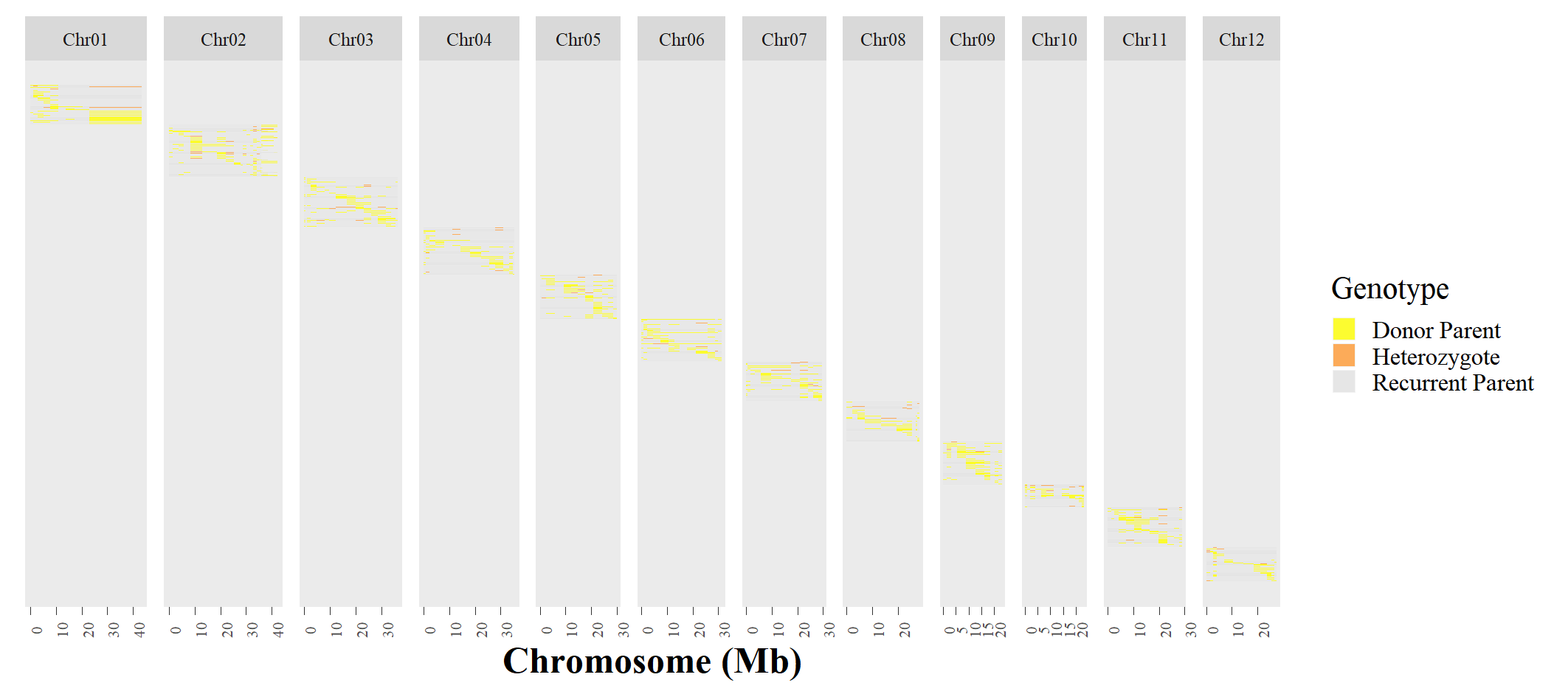

Supplement: Supplementary Figure 8 — Chromosome segments distribution and coverage degree of introgression library from the donor of upland rice. Each block at the row represented introgression from a total of 731 ILs on the target chromosome, regardless of introgression segments in other chromosomes, and each column represented a molecular marker locus. [file Image_8.TIFF]
